# Hyperkalemia and Pneumonia: A Retrospective Study on Mortality Outcomes in Southwest Missouri

**DOI:** 10.7759/cureus.78565

**Published:** 2025-02-05

**Authors:** Sukhmanjit Kaur, Alexandra Belohlavek, Melissa Bryan, Celine Nguyen, Scott A Andelin, Nova Beyersdorfer, Kerry Johnson, John Paulson

**Affiliations:** 1 Department of Primary Care, Kansas City University College of Osteopathic Medicine, Joplin, USA; 2 Department of Mathematics, Missouri Southern State University, Joplin, USA; 3 Department of Family Medicine, Freeman Health Systems, Joplin, USA

**Keywords:** electrolyte abnormality, hospitalization, hyperkalemia, mortality, pneumonia

## Abstract

Background: Pneumonia stands as a widely known contributor to hospitalization and mortality among adults in the United States. Meanwhile, disruptions in potassium homeostasis such as hyperkalemia may have an impact on in-patient mortality. This study seeks to examine the presence of hyperkalemia and its association with in-patient mortality among pneumonia patients.

Methods: Electronic medical records were used to perform a retrospective observational cohort study in Southwest Missouri patients admitted to the hospital with pneumonia and/or hyperkalemia. Patients were divided into three samples: patients with pneumonia and hyperkalemia (P1), pneumonia without hyperkalemia (P2), and hyperkalemia without pneumonia (P3). The goal was to determine and compare the in-patient mortality rates of these samples.

Results: Patients with both pneumonia and hyperkalemia (P1) demonstrated the highest mortality rate, ranging from 34.25% to 42.31%, significantly surpassing rates observed in patients with pneumonia without hyperkalemia (P2) or hyperkalemia without pneumonia (P3). Notably, patients with pneumonia without hyperkalemia (P2) exhibited a mortality rate comparable to patients with hyperkalemia without pneumonia (P3).

Conclusion: Our study revealed that patients admitted to the hospital with pneumonia and hyperkalemia had a statistically significant increase in mortality in comparison to patients with pneumonia or hyperkalemia independently. Recognizing this association may help identify prognosis and thus guide the management of patients admitted to the hospital with both hyperkalemia and pneumonia.

## Introduction

In the United States, pneumonia is known to be an important cause of hospitalization and mortality [1]. Mortality rates in patients hospitalized with pneumonia may be as high as 41% in patients admitted to the ICU [2]. Prior studies have recognized respiratory failure, chronic heart failure, dialysis, and sepsis as risk factors for death in patients with pneumonia [3,4]. 

Disruption of potassium homeostasis, as seen in hyperkalemia, is associated with life-threatening cardiac dysrhythmias and in-hospital mortality [4,5]. Common risk factors for hyperkalemia include chronic heart failure, chronic kidney disease, hypertension, and drugs [6,7]. Furthermore, an increase in all-cause mortality in association with hyperkalemia has been demonstrated by large-scale observational studies [6,8,9]. 

There has been some evidence that suggests that patients with certain respiratory infections have worse outcomes if hyperkalemia is present. Amin et al. reported that among patients with COVID-19, the presence of hyperkalemia was associated with higher in-hospital mortality compared to non-hyperkalemia patients, independent of the severity of illness due to COVID-19 or other risk factors for hyperkalemia [10]. Despite established links between hyperkalemia and mortality across various hospitalized patients, limited data exists regarding its impact on pneumonia-specific outcomes. While our study did not evaluate specific causes of hyperkalemia or severity of pneumonia, it did aim to look at the presence of hyperkalemia and its overall association with in-patient mortality among patients with varying causes of pneumonia. This research was previously presented as a poster presentation at the Kansas City University (KCU) Research Symposium on April 3, 2024, in Joplin, MO, USA.

## Materials and methods

Data collection 

**Figure 1:**
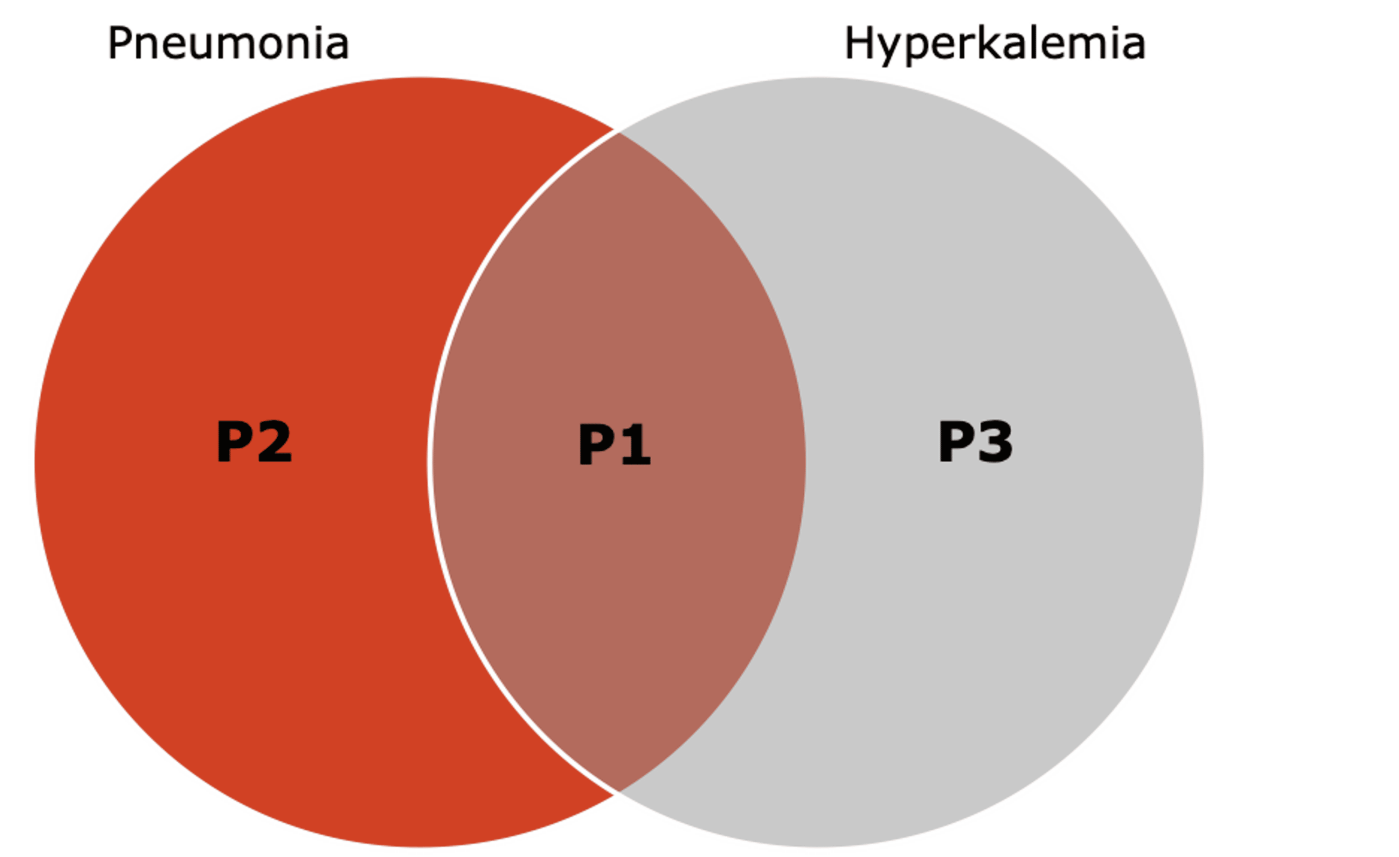
Classification of patient samples International Classification of Disease, Tenth Revision (ICD-10) codes were used to identify patients with pneumonia without hyperkalemia (P2), pneumonia with hyperkalemia (P1), and hyperkalemia without pneumonia (P3).

This retrospective observational cohort study obtained electronic medical records (EMR) from Freeman Health System (FHS) in Joplin, MO, and Neosho, MO, located in Southwest Missouri, for analysis of patients admitted to the hospital. Data was obtained from January 1, 2019, to December 31, 2021. Electronic medical record (EMR) data was drawn from two initial samples of patients aged 18 years or older admitted to FHS. International Classification of Disease, Tenth Revision (ICD-10) codes for pneumonia (including various bacterial, viral, and other causes) and hyperkalemia were used to identify specific samples (Tables 1, 2) [11]. As data collection was based on ICD-10 code documentation within the EMR, specific cutoff values for hyperkalemia and specific causes of hyperkalemia were not utilized in the patient selection. The first sample selected included 4,414 patients with pneumonia. This sample was then subdivided into 599 patients with pneumonia and hyperkalemia (P1) and 3,855 patients with pneumonia but without hyperkalemia (P2). The second sample selected included 1,310 patients who had hyperkalemia but not pneumonia (P3) (Figure 1). Patient identifiers were removed to keep patient confidentiality. This cohort study was retrospective and therefore did not require consent. 

**Table 1:** International Classification of Disease, Tenth Revision (ICD 10) codes utilized to identify patients with pneumonia Data was pulled from the electronic medical record (EMR) at Freeman Health System (FHS) in Joplin, MO, and Neosho, MO, and applied to patients admitted to the hospital from January 1, 2019, to December 31, 2021. Patients with any cause of pneumonia including bacterial, viral, fungal, or other were included in the pneumonia sample groups.

Pneumonia ICD-10 Codes	Diagnosis
J1000	Influenza due to other identified influenza virus with unspecified type of pneumonia
J1001	Influenza due to other identified influenza virus with the same identified influenza virus pneumonia
J1008	Influenza due to other identified influenza virus with other specified pneumonia
J1100	Influenza due to an unidentified influenza virus with an unspecified type of pneumonia
J1108	Influenza due to an unidentified influenza virus with specified pneumonia
J120	Adenoviral pneumonia
J121	Respiratory syncytial virus pneumonia
J122	Parainfluenza virus pneumonia
J123	Human metapneumovirus pneumonia
J1281	Pneumonia due to SARS-associated coronavirus
J1282	Pneumonia due to coronavirus disease 2019
J1289	Other viral pneumonia
J129	Viral pneumonia, unspecified
J13	Pneumonia due to Streptococcus pneumoniae
J14	Pneumonia due to Hemophilus influenzae
J150	Pneumonia due to Klebsiella pneumoniae
J151	Pneumonia due to Pseudomonas
J1520	Pneumonia due to Staphylococcus, unspecified
J15211	Pneumonia due to methicillin-susceptible Staphylococcus aureus
J15212	Pneumonia due to methicillin-resistant Staphylococcus aureus
J1529	Pneumonia due to other Staphylococcus
J153	Pneumonia due to Streptococcus, group B
J154	Pneumonia due to other streptococci
J155	Pneumonia due to Escherichia coli
J156	Pneumonia due to other Gram-negative bacteria
J157	Pneumonia due to Mycoplasma pneumoniae
J158	Pneumonia due to other specified bacteria
J159	Unspecified bacterial pneumonia
J168	Pneumonia due to other specified infectious organisms
J17	Pneumonia in diseases classified elsewhere
J180	Bronchopneumonia, unspecified organism
J181	Lobar pneumonia, unspecified organism
J188	Other pneumonia, unspecified organism
J189	Pneumonia, unspecified organism
J84116	Cryptogenic organizing pneumonia
J851	Abscess of lung with pneumonia
J95851	Ventilator-associated pneumonia

**Table 2:** International Classification of Disease, Tenth Revision (ICD 10) code utilized to identify patients with hyperkalemia Data was pulled from the electronic medical record (EMR) at Freeman Health System (FHS) in Joplin, MO, and Neosho, MO, and applied to patients admitted to the hospital from January 1, 2019, to December 31, 2021.

Hyperkalemia ICD-10 Code	Diagnosis
E875	Hyperkalemia

Data analysis 

The goal of the analysis was to determine whether patients admitted to the hospital with pneumonia and hyperkalemia have higher mortality rates than patients with pneumonia but no hyperkalemia and patients with hypokalemia but no pneumonia. The mortality rate was defined as the proportion of each sample that died while in the hospital. The data was analyzed using two-sample proportion tests using Wald’s method. The data was considered significant when p < 0.05. A 95% confidence interval for proportion difference was also used. Confounding variables were age, sex, ethnicity, and other concurring medical conditions. However, by comparing patients from the same regional area, these confounding variables are potentially limited. 

## Results

Of the 559 patients with pneumonia with hyperkalemia (P1), 214 expired during the hospitalization. Of the 3,855 patients with pneumonia without hyperkalemia (P2), 571 expired during the hospitalization. Of the 1,310 patients with hyperkalemia without pneumonia (P3), 204 expired during the hospitalization. The sample group P1 demonstrated the highest in-hospital mortality rate, which was 214 out of 559 (38.28% with 95% CI: 34.25% to 42.31%), significantly surpassing P2, which had a mortality rate of 571 out of 3,855 (14.81% with 95% CI: 13.69% to 15.93%), and P3, which had a mortality rate of 204 out of 1,310 (15.57% with 95% CI: 13.61% to 17.54%) as demonstrated in Table 3.

**Table 3:** Sample patient groups Patients were divided into those with pneumonia with hyperkalemia (P1), pneumonia without hyperkalemia (P2), and hyperkalemia without pneumonia (P3). Numbers of patients per group (n) are listed along with mortality rates as defined as percentage of patients that died during the hospitalization. A 95% confidence interval (CI) for mortality is included.

Sample Groups	n	Deaths	Mortality (%)	95% CI for Mortality (%)
Pneumonia with hyperkalemia (P1)	559	214	38.28	34.25 - 42.31
Pneumonia without hyperkalemia (P2)	3855	571	14.81	13.69 - 15.93
Hyperkalemia without pneumonia (P3)	1310	204	15.57	13.61 - 17.54

**Figure 2:**
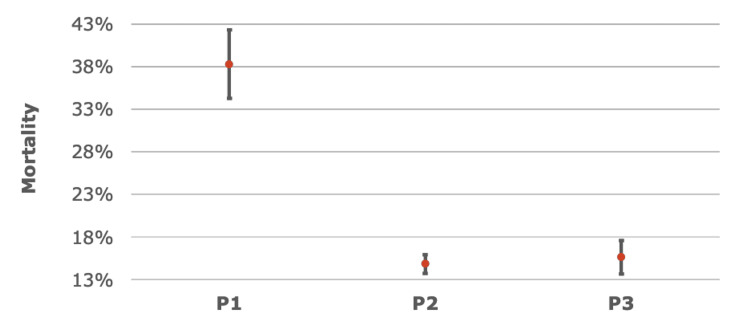
Confidence intervals (CI) of the mortality rates across each patient sample Patients with both pneumonia and hyperkalemia (P1) demonstrated the highest mortality rate, ranging from 34.25% to 42.31% based on 95% CI, significantly surpassing rates observed in patients with pneumonia without hyperkalemia (P2) or hyperkalemia without pneumonia (P3). The mortality rate difference between P1 and P2 was 23.47% (p <0.05, 95% CI (19.29%, 27.65%)), and between P1 and P3 was 22.71% (p <0.05, 95% CI (18.23%, 27.19%)). The data was analyzed using two-sample proportion tests using Wald’s method with Z-stat used to calculate p-values.

The mortality rate of P1 was significantly higher than P2 (p < 0.0001), and the difference in mortality rates between these two groups was 23.47% (95% CI: 19.29%-27.65%). Patients in P1 also had a significantly higher mortality rate when compared with P3 (p < 0.0001), with the difference in mortality rates being 22.71% (95% CI: 18.23%-27.19%). There was no significant difference in mortality rate between P2 and P3 (p = 0.5054) (Table 4, Figure 2).

**Table 4:** Mortality confidence intervals (CI) in two sample comparisons between sample groups with pneumonia and hyperkalemia P1: pneumonia with hyperkalemia; P2: pneumonia without hyperkalemia; P3: hyperkalemia without pneumonia. Sample 1 mortality corresponds to the first sample group listed under Sample Group Comparison for each row, while Sample 2 mortality corresponds to the second sample group listed under Sample Group Comparison for each row, respectively. The data was analyzed using two sample proportion tests using Wald’s method.

Sample Group Comparison	Sample 1 Mortality (%)	Sample 2 Mortality (%)	Sample 1 vs. Sample 2 (%)	Lower 95% CI for P1-P2	Upper 95% CI for P1-P2	Z-Stat	p-value
P1 vs. P2	38.28	14.81	23.47	19.29	27.65	13.5623	< 0.0001
P1 vs. P3	38.28	15.57	22.71	18.23	27.19	10.7881	< 0.0001
P2 vs. P3	14.81	15.57	0.76	-	-	-0.6660	0.5054

Therefore, the mortality rate in the sample with pneumonia and hyperkalemia (P1) was significantly higher than in patients with pneumonia or hyperkalemia without the other (P2, P3). Notably, patients with pneumonia without hyperkalemia (P2) exhibited a mortality rate similar to that of patients with hyperkalemia without pneumonia (P3), underscoring a distinct impact when both conditions coexist.

## Discussion

Pneumonia can exhibit varying degrees of severity and continues to be a common cause of hospitalization [1]. Pneumonia can be caused by several pathogens, bacteria being most common but also potentially by viruses or less commonly fungi. The diagnoses classified as pneumonia in the ICD-10 can be seen in Tables 1, 2. It is evident that pneumonia itself is a significant cause of mortality [2]. Studies have looked are risk factors for increased mortality in patients admitted to the hospital with pneumonia; some of these risk factors include male sex, high Acute Physiology and Chronic Health Evaluation (APACHE) II scores, and chronic heart failure [3]. 

Hyperkalemia frequently indicates an emergent state of electrolyte imbalance that may exacerbate cardiac and renal conditions. There is strong evidence that hyperkalemia is associated with an increased rate of patient mortality [5]. In acute hyperkalemia, potassium is a major intracellular cation, capable of lowering cell membrane potential and thus causing cardiac arrhythmias and neuromuscular complications. While chronic hyperkalemia may not always pose immediate adverse consequences, its association with chronic diseases (e.g., chronic kidney disease, cardiovascular disease, diabetes mellitus) may increase the risk of mortality [6,7]. 

The purpose of this study was to evaluate whether hyperkalemia is associated with a change in in-hospital mortality rates in patients admitted to the hospital with pneumonia. We found a statistically significant increase in mortality rates among pneumonia patients with a secondary diagnosis of hyperkalemia (P1) when compared to patients with pneumonia but no hyperkalemia (P2) and those with hyperkalemia but no pneumonia (P3). These findings suggest that the presence of hyperkalemia is associated with increased mortality in patients admitted to the hospital with pneumonia. However, as the mortality rate of patients with pneumonia without hyperkalemia (P2) was found to be similar to those with hyperkalemia without pneumonia (P3), it can also be suggested that the presence of pneumonia is associated with increased mortality in patients admitted to the hospital with hyperkalemia. 

Limitations

There are several limitations to this study. There was no use of a Pneumonia Severity Index (PSI) or any way of measuring the severity of the condition. Similarly, the degree of hyperkalemia as well as its underlying cause was not considered. Due to the method of utilizing hospital ICD-10 codes for the identification and selection of patients, the study did not differentiate between acute versus chronic hyperkalemia. In addition, the study did not distinguish between patients with pneumonia and/or hyperkalemia present upon admission to the hospital versus those who developed pneumonia and/or hyperkalemia during the hospitalization. There may have been other comorbid conditions not assessed in this study that may affect a patient’s mortality. Other factors that were not evaluated include social determinants of health such as socioeconomic status, education, employment, and environment.

In addition, the sample was not chosen at random and therefore may not be representative of the population. The sample was collected from Freeman Hospital in Joplin and Neosho, MO which serves the population of Southwest Missouri, along with its neighboring states, Arkansas, Oklahoma, and Kansas. The restricted sample size limits our discussion to those who reside in this region and experience barriers to health that are specific to rural communities [12]. The sample size in the Midwest is primarily Caucasian population; therefore, results may not be generalized to reflect a wider population. Furthermore, this study heavily relied on accurate physician documentation and thus only studied the sample in which the two diagnoses were recorded.

## Conclusions

Our study revealed that patients admitted to the hospital with pneumonia and hyperkalemia exhibited a statistically significant increase in mortality, in comparison to patients with pneumonia or hyperkalemia independently. In patients with pneumonia, a secondary diagnosis of hyperkalemia was associated with increased mortality. Conversely, in patients with hyperkalemia, pneumonia was associated with increased mortality. While a causal relationship cannot be determined based on this retrospective study, recognizing this association of increased mortality when patients are admitted to the hospital with both pneumonia and hyperkalemia may help with determining the prognosis of patients. Further studies may be considered to look at other factors, such as disease severity, sex, additional comorbidities, and social determinants of health, that may be contributing to this increased mortality.

## References

[1] Mattila JT, Fine MJ, Limper AH, Murray PR, Chen BB, Lin PL (2014). Pneumonia. Treatment and diagnosis. Ann Am Thorac Soc.

[2] Peters ZJ, Ashman JJ, Schwartzman A, DeFrances CJ (2022). National Hospital Care Survey demonstration projects: examination of inpatient hospitalization and risk of mortality among patients diagnosed with pneumonia. Natl Health Stat Report.

[3] Li G, Cook DJ, Thabane L (2016). Risk factors for mortality in patients admitted to intensive care units with pneumonia. Respir Res.

[4] Hunter RW, Bailey MA (2019). Hyperkalemia: pathophysiology, risk factors and consequences. Nephrol Dial Transplant.

[5] Jain N, Kotla S, Little BB, Weideman RA, Brilakis ES, Reilly RF, Banerjee S (2012). Predictors of hyperkalemia and death in patients with cardiac and renal disease. Am J Cardiol.

[6] Hougen I, Leon SJ, Whitlock R, Rigatto C, Komenda P, Bohm C, Tangri N (2021). Hyperkalemia and its association with mortality, cardiovascular events, hospitalizations, and intensive care unit admissions in a population-based retrospective cohort. Kidney Int Rep.

[7] Hoppe LK, Muhlack DC, Koenig W, Carr PR, Brenner H, Schöttker B (2018). Association of abnormal serum potassium levels with arrhythmias and cardiovascular mortality: a systematic review and meta-analysis of observational studies. Cardiovasc Drugs Ther.

[8] Kovesdy CP, Matsushita K, Sang Y (2018). Serum potassium and adverse outcomes across the range of kidney function: a CKD Prognosis Consortium meta-analysis. Eur Heart J.

[9] Ravioli S, Gygli R, Funk GC, Exadaktylos A, Lindner G (2021). Prevalence and impact on outcome of sodium and potassium disorders in patients with community-acquired pneumonia: a retrospective analysis. Eur J Intern Med.

[10] Amin A, Moon R, Agiro A, Rosenthal N, Brown H, Legg R, Pottorf W (2022). In-hospital mortality, length of stay, and hospitalization cost of COVID-19 patients with and without hyperkalemia. Am J Med Sci.

[11] (2022). ICD 10: International Classification of Diseases (ICD-10). https://www.cdc.gov/nchs/icd/icd-10-cm/index.html.

[12] Dauner KN, Loomer L (2021). A qualitative assessment of barriers and facilitators associated with addressing social determinants of health among members of a health collaborative in the rural Midwest. BMC Health Serv Res.

